# Microbial Contamination in Contact Lenses, Lens Care Solutions, and Accessories Among Asymptomatic Soft Contact Lens Users

**DOI:** 10.7759/cureus.66682

**Published:** 2024-08-12

**Authors:** Agnė Kirkliauskienė, Rūta Vosyliūtė, Viktorija Belousova, Marija Jakubauskienė, Petras Purlys, Laura Nedzinskienė, Ho Yiu Sung

**Affiliations:** 1 Department of Physiology, Biochemistry, Microbiology, and Laboratory Medicine, Faculty of Medicine, Institute of Biomedical Sciences, Vilnius University, Vilnius, LTU; 2 Faculty of Medicine, Vilnius University, Vilnius, LTU; 3 Department of Public Health, Faculty of Medicine, Institute of Health Science, Vilnius University, Vilnius, LTU; 4 Department of Artificial Intelligence, Artificial Intelligence Association of Lithuania, Vilnius, LTU; 5 Department of Anatomy, Histology, and Anthropology, Faculty of Medicine, Institute of Biomedical Sciences, Vilnius University, Vilnius, LTU

**Keywords:** hygiene skills, compliance, microbial contamination, asymptomatic soft contact lens wearers, contact lens

## Abstract

Objective

This study aimed to evaluate microbial contamination of contact lenses (CL) and their accessories among asymptomatic lens users and identify behavioral risk factors that might exacerbate the said contamination.

Methodology

Ninety-five asymptomatic soft CL users were recruited. In total, 380 samples were collected from the inner surface of lenses, the base of lens cases, the tip of the multipurpose solution bottle, and the solution itself. All swabs with samples were inoculated onto Columbia 5% sheep blood agar, MacConkey agar, Pseudomonas agar with cetrimide, and Sabouraud dextrose agar. Blood agar, MacConkey agar, and Pseudomonas agar with cetrimide were incubated at 37 °C for 24-48 hours. Fungal growth was investigated on Sabouraud dextrose agar, incubated at 25 °C, and examined daily for three weeks. Microscopic examination, culture-based methods, and biochemical tests were used to identify isolated microorganisms. A self-administered questionnaire on compliance with care and hygiene procedures was completed by each participant.

Results

The overall microbial contamination of tested samples was 38.7%. The most frequently contaminated items were lens cases (59, 62.1%), followed by bottles (44, 46.3%) and lenses (35, 36.8%). Meanwhile, the lowest incidence of contamination was seen in lens multipurpose solutions (9, 9.5%). The predominant microorganisms recovered were Coagulase-negative Staphylococci (CoNS) (94, 64%) and Gram-positive rods (29, 19.7%). Other identified potential pathogens were *Staphylococcus aureus *(11, 7.5%), *Pseudomonas aeruginosa *(5, 3.4%), *Escherichia coli *(1, 0.7%), and *Candida albicans *(2, 1.4%). The questionnaire revealed that contact lens users aged 18 to 20 showed a lack of compliance with proper hygienic care for contact lens maintenance. Risk factors such as male gender, smoking, showering, or swimming while wearing CL were related to microbiological contamination in at least one of the samples (*P* > 0.05).

Conclusions

The highest degree of contamination with highly virulent pathogens was determined in lens cases owing to insufficient lens care practices among study participants. Noncompliance with the lens cleaning procedures can lead to microbial colonization of the lens and its accessories, prompting inflammatory events in the eyes in the future.

## Introduction

Contact lenses (CL) have become increasingly popular since their inception, famously used by Leonardo da Vinci [1] in the sixteenth century to correct refractive errors. Over the past century, they have been prescribed extensively for optical, medical, and cosmetic purposes. CL offer numerous advantages, including enhanced peripheral vision, weather resistance, and heightened comfort for athletes. However, their widespread use is accompanied by a myriad of complications, such as CL-related acute red eye, corneal ulcers, and microbial or infiltrative keratitis, which can severely impair visual acuity [2-4].

Inflammation from CL use can be caused by bacteria, scratched corneas [3], hypoxia, changes in the tear film, and mechanical trauma [1]. Studies have shown that wearing CL alters the ocular microbiota [2]. When CL are inserted, proteins, glycoproteins, and lipids quickly build up, creating a conducive environment for bacterial growth [1,2]. Proper treatment requires distinguishing between infectious and noninfectious etiologies of the pathology [1].

The increased use of CL is partly due to their high accessibility. They can be commonly found over the counter, in optical outlets, online, and even in vending machines and can be attained without a prescription or consultation with an optometrist or ophthalmologist [5]. Those who purchase CL from these sources may not receive the thorough eye examinations and education provided in eye clinics and optometric centers.

Contamination of CL and lens care equipment is mostly due to noncompliance with CL care guidelines [5-7]. Failing to follow lens cleaning procedures increases the risk of microbial contamination, leading to conditions like keratitis and irreversible corneal damage. Noncompliance rates range from 40% to 91% [8] and are higher among young people aged 15-25 years, who are at greater risk for eye-related inflammatory events [9]. Many CL wearers diagnosed with corneal erosions during health visits do not show other infection symptoms [3], which might be attributed to inadequate knowledge of CL care guidelines, lack of risk awareness, or intentional disregard of recommendations [9]. Improving CL wearers' knowledge and hygiene practices is crucial for prevention [10].

The main aim of this study was to determine the degree and prevalence of microbial contamination in CL and its accessories, identify the contaminants, and assess self-reported CL-wearing behavioral risk factors among CL users.

## Materials and methods

Participant selection

The target population was asymptomatic, healthy local Vilnius University undergraduates who had been using soft CL and multipurpose solutions regularly (for more than one month) and had given their informed consent for the study. A cluster sampling procedure was used. Collection of specimens from participants and self-administered survey were conducted in the Department of Microbiology, Institute of Biomedical Sciences, Vilnius University, from September 2022 to May 2023.

Specimen collection

CL cases and bottles of CL solution were collected. Specimens were taken from the concave, inner surface of lenses, the base of lens cases, multipurpose solution, and the tip of the solution bottle. A sterile cotton swab was pre-moistened with sterile distilled water and then lenses, cases, and the tip of the bottle were swabbed. Several drops of solution from the bottle were taken and transferred to a sterile tube. Four samples were taken for each research subject: CL, storage case, the tip of the bottle, and the solution itself.

Microbiological procedures (culturing and identification)

All samples were immediately vortexed in separate tubes containing saline solution and subsequently inoculated onto Columbia 5% sheep blood agar (Graso, Poland), MacConkey agar (Thermo Scientific Oxoid, United Kingdom), Pseudomonas agar with cetrimide (Thermo Scientific Oxoid, United Kingdom), and Sabouraud dextrose agar (Thermo Scientific Oxoid, United Kingdom). Blood agar, MacConkey agar, and Pseudomonas agar with cetrimide were incubated at 37 °C for 24-48 hours. Fungal growth was investigated on Sabouraud dextrose agar, incubated at 25 °C. The media were examined daily and discarded after three weeks if no fungal growth was observed, and such samples were registered as negative. The number of colonies and their morphologies were recorded for each inoculated medium. Gram staining and biochemical tests were performed to characterize bacterial isolates. For Gram-positive bacteria, tests included catalase, novobiocin disk, bacitracin, and coagulase tests. For Gram-negative bacteria, tests included triple sugar iron agar, indole, motility, citrate agar, lysine decarboxylase agar, and oxidase tests. Presumptive identification of *Candida albicans* was achieved through a combination of phenotypic methods. Isolates were initially cultured on Chromogenic *C. albicans* agar (Thermo Scientific Oxoid, United Kingdom), where the formation of characteristic green colonies was indicative of *C. albicans*. Subsequently, the germ tube test was performed as a confirmatory step, with the presence of germ tubes providing further evidence of *C. albicans *identification.

Research instrument

The 17-item research instrument, developed by researchers, comprised three sections: (1) sociodemographic variables (e.g., age and gender); (2) risk factors for microbial keratitis development (e.g., duration, frequency, and purpose of contact lens use, smoking); and (3) behavioral patterns and hygiene practices (e.g., handwashing, lens rubbing, solution replacement, bottle closure, leaving the case to air dry upside down, frequency of changing cases, using lenses past their expiration date, and sleeping with CL). The latter sections were informed by the Centers for Disease Control and Prevention (CDC, United States) guidelines on recommended behaviors for contact lens wearers [11]. Respondents' hygiene skills were evaluated as either sufficient (hygiene skills score of 5-9) or insufficient (hygiene skills score of 0-4) (Appendix).

Data analysis

Microbial contamination was evaluated in relation to the steps of the contact lens care routine using the Fisher Exact test. Statistical significance was determined at *P* < 0.05 for all tests. Risk factors were assessed by calculating the odds ratio (OR) and 95% confidence intervals (CI). Data analysis was performed using SPSS for Windows, Version 24.0 (IBM Corp., Armonk, NY).

Ethical approval

The ethical approval for the study was obtained from the regional biomedical ethics review board (approval no. 158200-15-784-300). Written informed consent was received from all participants.

## Results

A total of 95 individuals were recruited for this study: 78 (82.1%) were female and 17 (17.9%) were male. The mean age of the participants was 20.5 years (standard deviation [SD] = 1.32), with an age range of 18-25 years. Nearly all participants (93, 97.9%) wore CL for optical purposes, primarily for the correction of refractive errors such as myopia, hyperopia, and astigmatism.

A total of 380 specimens were collected from asymptomatic research subjects. The overall microbial contamination rate was 38.7% (147; 95% CI 32.5-44.9). The most commonly contaminated items were lens cases, with a contamination rate of 62.1% (59; 95% CI 58.3-65.9), followed by bottles at 46.3% (44; 95% CI 40.9-51.7), and lenses at 36.8% (35; 95% CI 30.4-43.1). The lowest incidence of contamination was observed in lens multipurpose solution, at 9.5% (9; 95% CI 0.4-18.6).

Predominant microorganisms included CoNS (94, 64.0%) and Gram-positive rods (29, 19.7%) (Table 1). Other identified potential pathogens included *S. aureus* (11, 7.5%), *P. aeruginosa *(5, 3.4%), *E. coli* (1, 0.7%), and *C. albicans *(2, 1.4%). Highly virulent pathogens such as *S. aureus* (5, 5.3%) and *P. aeruginosa *(4, 4.2%) were the most frequently isolated microorganisms from lens cases. *C. albicans* was detected in samples from bottle tips and solutions. The majority of samples (92.1%) exhibited polymicrobial growth of microorganisms.

**Table 1:** Frequencies of various microorganisms isolated from the contaminated samples of asymptomatic CL wearers (N = 147). *n*, number of samples; CL, contact lenses

Isolated microorganisms	Contact lens (*n* = 95)		Lens case (*n* = 95)		Tip of the bottle (*n* = 95)		Multipurpose solution (*n* = 95)		In total (*N* = 147)	
	n	%	n	%	n	%	n	%	n	%
*Staphylococcus aureus *	2	2.1	5	5.3	4	4.2	0	0.0	11	7.5
CoNS	20	21.1	44	46.3	27	28.4	3	3.2	94	64.0
*Micrococcus* spp.	4	4.2	4	4.2	7	7.4	0	0.0	15	10.2
*Streptococcus* spp.	4	4.2	9	9.5	2	2.1	0	0.0	15	10.2
Pseudomonas aeruginosa	0	0.0	4	4.2	0	0.0	1	1.1	5	3.4
*Bacillus* spp.	1	1.1	0	0.0	1	1.1	0	0.0	2	1.4
*Escherichia*​​​​​​​* coli *	0	0.0	0	0.0	1	1.1	0	0.0	1	0.7
*Candida*​​​​​​​* albicans *	0	0.0	0	0.0	1	1.1	1	1.1	2	1.4
Other Gram-positive rods	2	2.1	13	13.7	11	11.6	3	3.2	29	19.7
Other Gram-negative rods	1	1.1	5	5.3	6	6.3	4	4.2	16	10.9
Gram-negative cocci	2	2.1	5	5.3	5	5.3	1	1.1	13	8.8

The study results were assessed according to the lens care skills level (sufficient/insufficient). Insufficient hygiene skills (score 0-4) were observed in 35 participants (36.8%). The study indicated that specifically 18- to 20-year-old males who wear CL occasionally demonstrated a higher risk of having insufficient hygiene skills. A statistically significant difference was, however, not detected (Table 2). Insufficient lens care skills increased the risk of microbial contamination in all collected specimens. Nevertheless, the risk of microbiological contamination with 4 to 17 different microbial strains appeared to be lower in this research group.

**Table 2:** Association between CL care skills and various demographic, social and microbiological factors. Statistical significance was determined at P < 0.05. *n*, number of samples; OR, odds ratio; CI, confidence interval; CL, contact lenses

Factors	Total	Lens care skills group (*n* = 95)		OR (95% CI)	*P*-value
		Insufficient skills (*n* = 35)	Sufficient skills (*n* = 60)		
	*n* (% in the study group)	*n* (% in the skills group and % in the study group)	*n* (% in the skills group and % in the study group)		
Gender					
Male	17 (17.9)	7 (20.0, 7.3)	10 (10.7, 10.5)	1.25 (0.43-3.65)	0.783
Female	78 (82.1)	28 (80.0, 29.5)	50 (83.3, 52.7)		
Age					
18-20 years	15 (15.8)	6 (17.1, 6.3)	9 (15.0, 9.5)	1.17 (0.38-3.63)	0.778
21-25 years	80 (84.2)	29 (82.9, 30.5)	51 (85.0, 53.7)		
Frequency of wearing lenses per week					
daily	68 (71.6)	23 (65.7, 24.2)	45 (75.0, 47.4)		
3-4 times a week	15 (15.8)	5 (14.3, 5.2)	10 (16.7, 10.5)	7.83 (0.83-74.12)	0.245
1-2 times a week	7 (7.4)	3 (8.6, 3.2)	4 (6.7, 4.2)		
on special occasions	5 (5.2)	4 (11.4, 4.2)	1 (1.6, 1.1)		
Contamination					
At least 1 sample	77 (81.1)	30 (85.7, 31.6)	47 (78.3, 49.5)	1.66 (0.54-5.13)	0.429
All samples	18 (18.9)	5 (14.3, 5.2)	13 (21.7, 13.7)		
Contact lenses					
Contaminated	35 (36.8)	16 (45.7, 16.8)	19 (31.7, 20.0)	1.82 (0.77-4.29)	0.192
Uncontaminated	60 (63.2)	19 (54.3, 20.0)	41 (68.3, 43.2)		
Storage case					
Contaminated	59 (62.1)	22 (62.9, 23.2)	37 (61.7, 38.9)	1.05 (0.44-2.49)	1.000
Uncontaminated	36 (37.9)	13 (37.1, 13.7)	23 (38.3, 24.2)		
Tip of the bottle					
Contaminated	44 (46.3)	17 (48.6, 17.9)	27 (45.0, 28.4)	1.15 (0.50-2.66)	0.832
Uncontaminated	51 (53.7)	18 (51.4, 18.9)	33 (55.0, 34.8)		
Multipurpose solution					
Contaminated	9 (9.5)	5 (14.3, 5.2)	4 (6.7, 4.2)	2.33 (0.58-9.35)	0.282
Uncontaminated	86 (90.5)	30 (85.7, 31.6)	56 (93.3, 59.0)		
Number of different species isolated from the participant samples					
From 4 to 17	48 (50.5)	17 (48.6, 17.9)	31 (51.7, 32.6)	0.88 (0.38-2.04)	0.833
From 0 to 3	47 (49.5)	18 (51.4, 18.9)	29 (48.3, 30.6)		

To assess the risks associated with demographic (gender) and behavioral factors (wearing lenses for more than two years, smoking, swimming, and showering while wearing lenses), respondents were put into two groups based on sample contamination status (not contaminated vs. contamination in one to four samples). The analysis revealed that male gender, smoking, and showering or swimming while wearing CL increased microbiological contamination in at least one sample (Table 3). Conversely, wearing CL for more than two years appeared to reduce the rate of contamination, although the difference was not statistically significant (*P* > 0.05).

**Table 3:** Association of gender and some behavioral risk factors with microbiological contamination. *n*, number of samples; OR, odds ratio; CI, confidence interval Statistical significance was determined at *P* < 0.05.

Factors	Contamination		OR (95% CI)	*P*-value
	Contaminated at least 1 in 4 samples (*n* = 77), *n* (%)	All samples were not contaminated (*n* = 18), *n* (%)		
Gender				
Male	15 (19.5)	2 (11.1)	1.94 (0.40-9.34)	0.514
Female	62 (80.5)	16 (88.9)		
Duration of lens wear				
More than 2 years	68 (88.3)	17 (94.4)	0.44 (0.05-3.75)	0.682
Less than 2 years	9 (11.7)	1 (5.6)		
Smoking				
Smoke	29 (37.3)	6 (33.3)	1.21 (0.41-3.57)	0.793
Don't smoke	48 (62.3)	12 (66.7)		
Swimming or showering while wearing lenses				
Yes	35 (45.5)	5 (27.8)	2.17 (0.71-6.67)	0.196
No	42 (54.5)	13 (72.2)		

Our study revealed that 30 (31.6%) of respondents did not wash their hands with soap before handling lenses, 38 (40%) did not replace the solution daily, and 24 (25.3%) added fresh solution to the existing one. Water exposure of lenses while swimming was reported by 40 participants (42.1%). A significant majority (74, 77.9%) reported not rinsing lenses with solution. Additionally, 78 (82.1%) of wearers exceeded the recommended lens replacement period. It was also noted that half of the participants, 48 (50.5%), admitted to sleeping while wearing lenses.

## Discussion

CL have seen increasing demand over recent decades due to the rising prevalence of refractive disorders and positive user experiences facilitated by advances in materials and technologies. Noncompliance with CL wearing recommendations significantly increases the risk of inflammatory eye diseases, particularly microbial keratitis [6,12,13]. Researchers are exploring CL designs incorporating antibacterial properties such as selenium, fimbrolides, the cationic peptide melamine, or silver ions [14]. Until antimicrobial CLs are developed, strategies to prevent contamination remain crucial.

In our study, 35 (36.8%) participants reported insufficient hygienic skills, which is reflected in an overall microbial contamination rate of 38.7% (147) in the samples. Similar studies also illustrated the correlation between poor compliance with care regimens and microbial contamination prevalence [14]. Higher compliance in our study may be attributed to the predominance of participants with medical education backgrounds.

Several studies have highlighted significantly higher rates of noncompliance and inadequate hygienic practices among CL users, increasing their susceptibility to eye infections [9,15-18]. However, some studies have not found a direct association between high noncompliance rates and microbial contamination [8].

Younger age, as well as extended wear of CL (day and night), has been identified as a risk factor for increasing the risk of developing eye-related infections by up to five times [9,19]. The eye possesses a powerful antibacterial system that can eliminate many organisms introduced during lens handling and implantation under normal circumstances [14]. For instance, most individuals are protected from corneal infections by their eye's innate defense mechanisms, such as the flushing action of tears and an intact corneal epithelium. However, extended wear of CL can disrupt these defenses, which may explain the higher rate of corneal infection associated with this type of lens usage [20].

In our study, the most contaminated item was lens cases (59, 62.1%), a finding consistent with other studies [21,22]. Lens cases provide a favorable environment for biofilm growth due to their static nature and low nutrient content. Their design, particularly the corners, makes them difficult to clean and susceptible to bacterial colonization [1], thereby increasing the incidence of microbial keratitis by up to four times [23-26]. The lowest contamination rate was detected in the solution, likely due to its ingredients (chlorhexidine gluconate and polyaminopropyl biguanide), which inhibit the bacterial reproduction cycle, particularly for *S. aureus* and *P. aeruginosa* [27].

Our findings reveal that the most predominant microorganism was CoNS (94, 64.0%). CoNS are generally considered commensal organisms. However, our data suggest (*P* = 0.196) that external water sources, such as swimming and showering while wearing CL, were not associated with potential pathogens. Potential pathogens for keratitis such as *S. aureus* and *P. aeruginosa* were isolated in 7.5% and 3.4% of cases, respectively. Identification of these microorganisms varies across studies, possibly due to differences in sampling or identification techniques. For instance, CoNS prevalence ranges from 21% to 42%, *P. aeruginosa* from 0 to 19.5%, and *S. aureus* from 8% to 21% of samples [3,5,14,16]. Notably, Raksha et al. did not exclude any of these species from asymptomatic contact lens wearers in their investigation [2].

The isolation rates of *Micrococcus* spp. (15, 10.2%), *Streptococcus* spp. (15, 10.2%), and *Bacillus* spp. (2, 1.4%) align with findings from other researchers [14,28,29]. In our study, we isolated *C. albicans* in 2 (1.4%) samples. Other studies often regard the isolation of fungi in lenses and their accessories as an unusual finding [2,5,14,30].

A key strength of this study lies in its novel contribution to the field, representing the first investigation of microbial contamination of CL, lens care solutions, and their accessories among asymptomatic soft CL users in Lithuania. This study establishes a foundation for future research and clinical practice in the country. A potential limitation of this study is that noncompliance was self-reported. Respondents may have either underreported their noncompliance or been overly critical of their behavior, classifying themselves as noncompliant even for relatively minor oversights. It is important to note that this study did not assess viral or protozoal contamination. Another limitation of this study is that the identification of the isolated bacteria relied on traditional culture methods. The inclusion of molecular methods could provide additional insights into the microbiological contamination of CL and their additives. Future studies should address the limitations of this research by incorporating molecular methods for a comprehensive microbial analysis and by assessing the presence of viral and protozoal contaminants in CL and their additives.

## Conclusions

One-third of the samples were microbially contaminated. The most commonly contaminated items were lens cases, bottles of solution, and lenses, while the least contaminated were multipurpose solutions (contaminated 1 in 10 samples). The majority of samples exhibited polymicrobial growth of microorganisms. The most predominant microorganisms included CoNS and Gram-positive rods. Other identified potential pathogens included *S. aureus*, *P. aeruginosa*, *E. coli*, and *C. albicans*. Highly virulent pathogens such as *S. aureus* and *P. aeruginosa* were the most frequently isolated microorganisms from lens cases. *C. albicans* was detected in samples from bottle tips and solutions. Male gender, smoking, and showering or swimming while wearing CL increased microbiological contamination in at least one sample.

Changing behavioral patterns in contact lens care routines, along with regular cleaning and replacement of accessories, should be noted as the most effective preventive measures. Future research and an evidence-based comprehensive set of guidelines are needed to enhance compliance with contact lens care hygiene standards among users.

## Appendices

Appendix

Questionnaire to assess self-reported contact lenses wearing behavioral risk factors among young people

1. Gender:

     o  Male

     o  Female

2. Age:    __________

3. For what purposes do you use contact lenses?

     o  Cosmetic

     o  Therapeutic

     o  Others:________________

4. Duration of use:

     o  >2 years

     o  <2 years

5. Frequency of lenses usage:

     o  Daily

     o  3-4 times per week

     o  1-2 times per week

     o  Occasional

6. Wearing contact lenses while smoking:

     o  Yes

     o  No

7. Wearing contact lenses while swimming/showering:

     o  Yes

     o  No

8. Are your contact lenses coloured?

     o  Yes

     o  No

9. Wearing contact lenses while sleeping:

     o  Yes

     o  No

10. Do you always wash your hands before putting in or removing contact lenses?

     o  Yes

     o  No

11. Do you always rub contact lenses before/after usage?

     o  Yes

     o  No

12. Do you always fully replace contact lens multipurpose solution from container daily?

     o  Yes

     o  No

13. Do you always close the bottle after the usage?

     o  Yes

     o  No

14. Do you always leave the container case to air dry upside down without the caps?

     o  Yes

     o  No

15. Do you change the container at least every 3 months?

     o  Yes

     o  No

16. Do you use contact lenses after their expiration date?

     o  Yes

     o  No

17. Do you top off contact lens solution?

     o  Yes

     o  No
